# Quadriceps and hamstring muscle activity during cycling as measured with intramuscular electromyography

**DOI:** 10.1007/s00421-016-3428-5

**Published:** 2016-07-23

**Authors:** Julio Cézar Lima da Silva, O. Tarassova, M. M. Ekblom, E. Andersson, G. Rönquist, A. Arndt

**Affiliations:** 1 The Swedish School of Sport and Health Sciences (GIH), Lidingövägen 1, Office: 1355, (Box: 5626), 114 86 Stockholm, Sweden; 2 School of Physical Education of the Federal University of Rio Grande do Sul (ESEF/UFRGS), Porto Alegre, Brazil; 3 Department of Neuroscience, Karolinska Institute, Stockholm, Sweden; 4 Department of CLINTEC, Karolinska Institute, Stockholm, Sweden

**Keywords:** Cycling, Intramuscular EMG, Muscle activation, Pedaling cycle, Thigh muscles

## Abstract

**Purpose:**

The aim of this study was to describe thigh muscle activation during cycling using intramuscular electromyographic recordings of eight thigh muscles, including the biceps femoris short head (BFS) and the vastus intermedius (Vint).

**Methods:**

Nine experienced cyclists performed an incremental test (start at 170 W and increased by 20 W every 2 min) on a bicycle ergometer either for a maximum of 20 min or to fatigue. Intramuscular electromyography (EMG) of eight muscles and kinematic data of the right lower limb were recorded during the last 20 s in the second workload (190 W). EMG data were normalized to the peak activity occurring during this workload. Statistical significance was assumed at *p* ≤ 0.05.

**Results:**

The vastii showed a greater activation during the 1st quadrant compared to other quadrants. The rectus femoris (RF) showed a similar activation, but with two bursts in the 1st and 4th quadrants in three subjects. This behavior may be explained by the bi-articular function during the cycling movement. Both the BFS and Vint were activated longer than, but in synergy with their respective agonistic superficial muscles.

**Conclusion:**

Intramuscular EMG was used to verify muscle activation during cycling. The activation pattern of deep muscles (Vint and BFS) could, therefore, be described and compared to that of the more superficial muscles. The complex coordination of quadriceps and hamstring muscles during cycling was described in detail.

## Introduction

Movement coordination in cycling is achieved by muscle recruitment and the associated modulation of musculotendinous architecture in the lower extremity (Li 2004). During pedaling, an optimization of quadriceps and hamstring muscle recruitment is desirable for producing power at the pedal. These muscle groups have been described as having different contributions at different phases of the pedal cycle, and different muscles within these groups can also be assumed to have different functional contributions. To better understand this contribution mechanism during pedaling, surface electromyography (EMG) has previously been used to measure muscle activation coordination in cycling (Prilutsky and Gregor 2000; Smirmaul et al. 2009; Soderberg and Cook 1984; Wakeling and Horn 2009).

Previous studies on the thigh muscles during cycling have shown that vastus lateralis (VL) and vastus medialis (VM) have maximum activation from approximately top dead center (TDC, 0°) of the pedal cycle to halfway (90°) through the propulsion phase (0–180°) (Dorel et al. 2008; Jorge and Hull 1986; Ryan and Gregor 1992). According to Savelberg and Meijer (2003), the vastii have an optimal muscle length at a flexed knee joint configuration (80.8°) and the RF is activated earlier than the vastii, which is presumably due to the bi-articular RF also flexing the hip joint (Ryan and Gregor 1992).

Previous studies report conflicting results on the activation of knee flexor activation. Ericson et al. (1985) showed activation of the semimembranosus (SemM) and semitendinosus (SemT) between 150° and 270° crank angle. Dorel et al. (2008) showed SemM activation during the downstroke phase together with the biceps femoris (both BFS and long head; BFL). Jorge and Hull (1986) showed a prolonged activity for the SemM between 60° and 240° of crank angle. However, Ryan and Gregor (1992) showed activation of the biceps femoris, SemM, and SemT during the complete pedaling cycle, with the highest activity during the downstroke phase (peak activation at 145° of crank angle) and minimum activation at 270° crank angle. Coactivation between knee extensors and flexors during the propulsion phase of pedaling has been suggested to regulate the net joint moments responsible for force transfer to the pedal (Hug et al. 2011; Jorge and Hull 1986; Li 2004; Ryan and Gregor 1992; So et al. 2005).

The surface EMG technique used in previous studies can be affected by crosstalk from adjacent muscles. This problem is especially pronounced when contracting muscles move relative to the skin and, therefore, also relative to the electrodes. Intramuscular EMG signals are less affected by crosstalk and provide greater accuracy and repeatability (Bogey et al. 2003; Jacobson et al. 1995). Intramuscular EMG also facilitates measurement of muscle activation in deep muscles with limited or no surface access, e.g., the Vint and ultrasound guided electrode insertion permits investigation of muscles with difficult surface access, such as the BFS (Andersson et al. 1997).

Previous studies have applied the intramuscular EMG technique in many different tasks (Andersson et al. 1997; Bogey et al. 2000; Bouisset and Maton 1972; Juker et al. 1998; Semciw et al. 2013). This technique is of interest in cycling, because flexible fine-wire electrodes can move with the muscle during pedaling even at high cadence (Chapman et al. 2010). Intramuscular EMG has been used previously to measure muscle activation patterns during cycling, but these studies have, to our knowledge, been restricted to investigations of the calf muscles (Chapman et al. 2006, 2007, 2008, 2009).

This study extends previous surface EMG investigations by providing information on superficial and deep quadriceps and hamstring activation during cycling. The aim of this study was to describe thigh muscle activation patterns during the pedaling cycle for experienced cyclists. This was the first study to use intramuscular EMG in four knee extensors and four knee flexors simultaneously during cycling. The results are expected to provide previously undisclosed information of how the central nervous system regulates muscle activation of deep thigh muscles (Vint and BFS) in synergy with superficial muscles (BFL, SemM, SemT, RF, VL, and VM) for cycling movement coordination.

## Methods

### Subjects

Nine subjects competitively active in either cycling or triathlon participated in this study. The sample characteristics were (mean ± SD): age 31.67 ± 10.87 years, weight 79.67 ± 4.72 kg, and height 182.67 ± 7.35 cm. The study was approved by the Stockholm regional ethical committee (approval nr: 2014/641-31/1), and informed consent was obtained from all participants.

### Protocol

Subjects warmed up on a bicycle ergometer (LC4, Monark Exercise AB, Sweden) for 10 min. After the warm-up, they performed an incremental test on the bicycle ergometer either for a maximum of 20 min or to fatigue. The initial workload of the cycling test was 170 W, and this was automatically increased by 20 W every 2 min by the Monark 939E analysis software (Monark Exercise AB, Sweden). The maximum workload at 20 min was, therefore, 350 W. The cyclists controlled their pedaling cadence at 90 ± 4 rpm using visual feedback from a monitor on the handlebars. Heart rate was measured by the Monark 939E analysis software (Monark Exercise AB, Sweden) throughout the test by a chest belt (Premium heart rate monitor, Garmin, USA). All data presented in this study were from the second workload (190 W) of the incremental test.

### Data collection

**Fig. 1:**
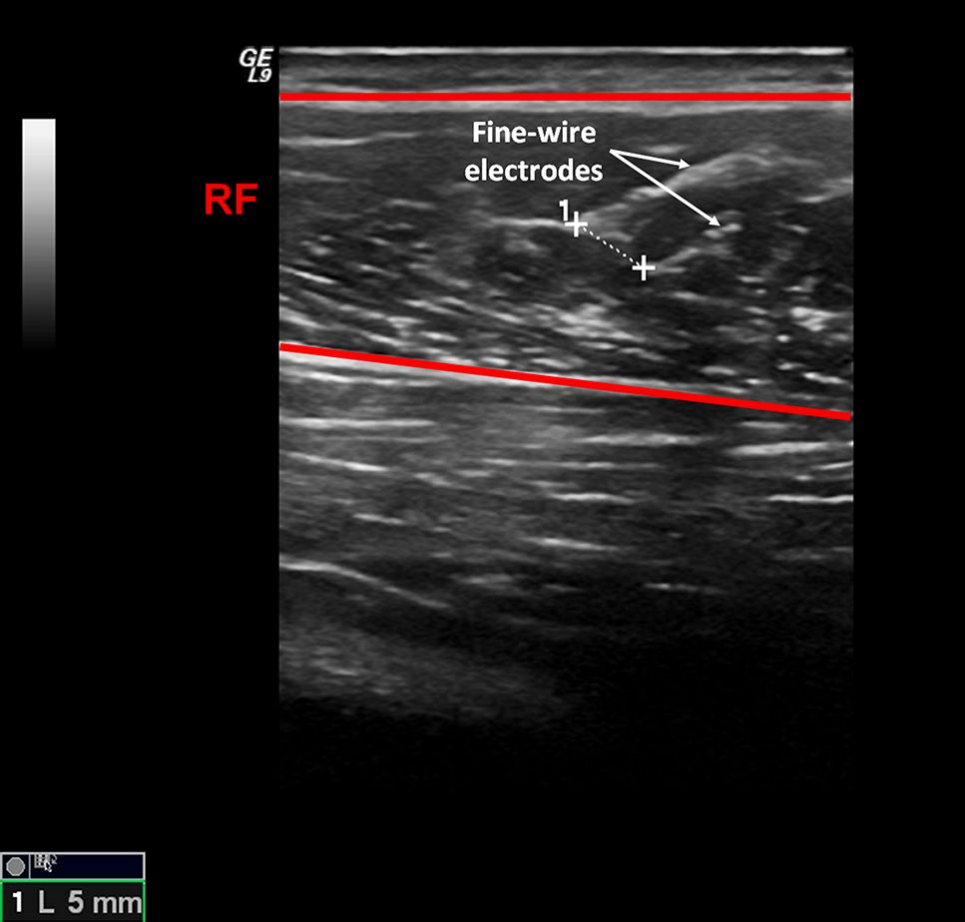
Ultrasound image acquired during fine-wire electrode insertion in the rectus femoris (RF) muscle (located between the *red lines*). The tips of the electrodes are marked with a *white cross*, indicating the inter-electrode distance (≈5 mm)

A fine-wire EMG system (MyoSystem 1400A, Noraxon Inc., USA) was used to measure the activation of all muscles in the right leg. BFS was only measured in four subjects due to a non-optimal electrode insertion location for the first five subjects, which resulted in unusable data. A linear 39 mm, 5–13 MHz probe [M12L, General Electric (GE), USA] connected to an ultrasound system (Logiq E9, GE, USA) sampling at 30 Hz was used for guiding insertion and verification of wire positions. Sixteen sterilized hypodermic needles (diameter 0.8 mm) were used to individually place each Teflon-coated, seven-stranded silver hook-wire electrode (diameter 0.25 mm with a stripped length of 3 mm) into the relevant muscle. Two fine-wire electrodes were percutaneously inserted into each muscle in a bipolar configuration with an inter-electrode distance of ≈5 mm (Fig. 1). A skin mounted reference electrode (Ambu, USA) was placed over an electrically neutral bony prominence (head of fibula). EMG signals were recorded at 5000 Hz per channel using a 16 bit, analog-to-digital converter [Power 1401, Cambridge Electronic Design (CED), England] in the Spike2 software (v7.0, CED, England). EMG data were recorded during the last 20 s of the 190 W workload.

Right lower limb kinematics were acquired using five optoelectronic cameras (Oqus 4-series, Qualisys AB, Sweden), recording at 250 frames per second via the Qualisys Track Manager (QTM^®^) software (Qualisys AB, Sweden). Spherical reflective markers (12 mm diameter) were placed on the acromion, anterior superior iliac spine, greater trochanter, thigh, lateral femoral condyle, shank, lateral malleolus, and center of crank, pedal, and on the shoe at the toe, heel, and fifth metatarsophalangeal joint. Kinematic data were recorded during the same 20 s period as the EMG data collection. The kinematic and EMG data were synchronized using an analog signal sent by the QTM software. An electrical switch on the bicycle frame registered each time the crank passed bottom dead center (BDC) and sent a 5 V trigger signal to the QTM and Spike2 systems. The plane formed by the rotating crank (center of crank and pedal markers) was calculated and defined as the global sagittal plane to correct for any deviation of the bicycle orientation relative to the calibrated global coordinate system. The crank angle was determined by the segment defined by the markers attached to the center of crank and pedal in the adjusted plane, and this angle was used for dividing the data into single, consecutive crank revolutions.

### Data analysis

**Fig. 2:**
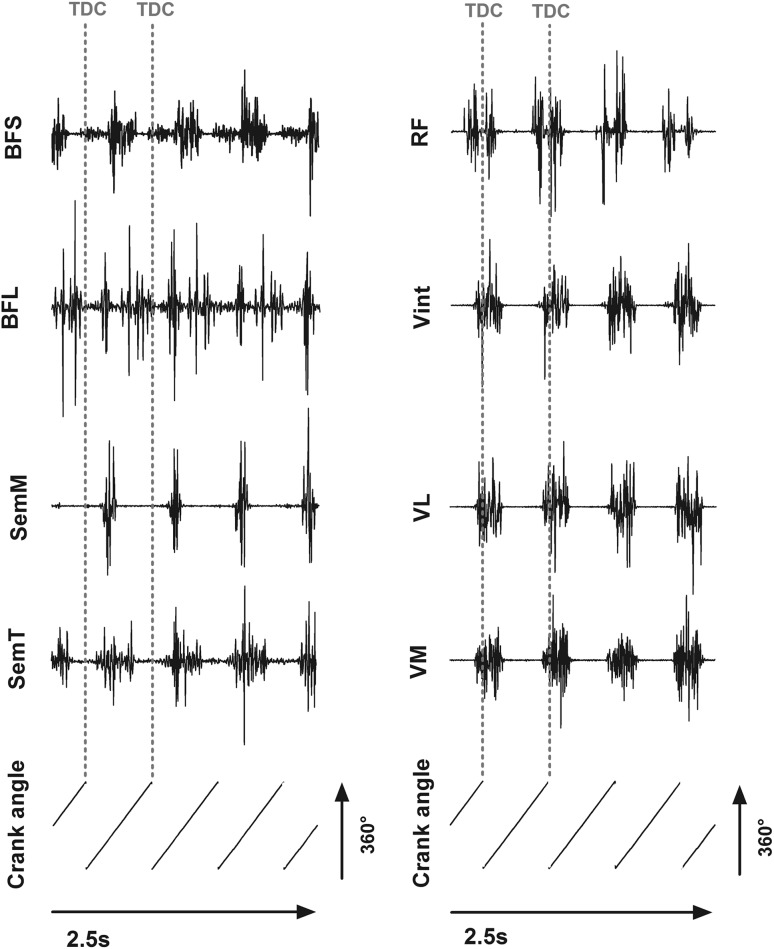
Representative raw EMG signals for hamstrings (*left*) and quadriceps (*right*) muscles and the crank angle signal from one subject. *Vertical red dotted lines* represent one individual pedaling cycle between two consecutive top dead centers (TDC)

Every muscle of every subject was analyzed for ten consecutive crank revolutions. The raw EMG signals (Fig. 2) were filtered using a band-pass Butterworth filter (50–1000 Hz) and a root-mean-square (RMS) envelope with a window of 20 ms was applied. The RMS data were normalized to the peak of the average RMS curves of ten consecutive crank revolutions, for each muscle of every subject. It has been shown that analyzing EMG amplitude during cycling has greater intraclass correlation coefficients if normalized to EMG peaks recorded during cycling rather than to maximum voluntary contractions (Albertus-Kajee et al. 2010).

The times of EMG onset and offset were identified from rectified and smoothed data. Onset and offset thresholds were defined as 20 % of the visually determined maximum activity, and a burst in muscle activation was identified when this level was maintained for ≥10 % of the crank cycle. EMG data analysis was conducted using the Spike2 (CED, England) software and in Matlab (Mathworks, USA).

The kinematic data were digitized and automatic tracking of markers was conducted in the QTM software. Kinematic data were smoothed with a digital second-order zero lag low-pass Butterworth filter with cut-off frequency of 10 Hz in the Visual 3D software (version 5.02, C-Motion Inc, USA). Hip, knee, and ankle joint angles were calculated from the smoothed *x*, *y,* and *z* coordinate data in the new sagittal plane determined by the crank rotation. Full-hip extension was defined as 180° and full-knee extension as 0°. Neutral position for the ankle joint was defined as 90°. All kinematic variables were averaged for ten consecutive crank revolutions for every subject.

The RMS EMG data and joint angles were divided into four quadrants (1st = 0–90°, 2nd = 90–180°, 3rd = 180–270°, and 4th = 270–360°) of the crank cycle, with 0° (360°) representing TDC. A division of the pedaling cycle into four crank quadrants has previously been used in the analysis of cycling biomechanics (Brown and Kautz 1998; Ting et al. 1998) and has been suggested to provide an easy indication of performance level at different pedal positions (Hasson et al. 2008). Other studies have, however, described muscle activation onset and offset relative to crank angle (Bieuzen et al. 2007; Chapman et al. 2006; Jobson et al. 2013), and the data in this study were, therefore, also presented in terms of activation onset and offset.

Individual means for all dependent variables over the ten crank cycles were calculated for each crank quadrant prior to averaging across all subjects. EMG peak magnitudes were acquired from the average curve from ten crank cycles of all subjects.

### Statistical analysis

Data are presented as means and standard deviations. The Shapiro–Wilk test and Mauchly tests were used to verify data normality and sphericity, respectively. The Greenhouse-Geisser correction factor was applied when the data did not show sphericity. To investigate how the EMG amplitude of individual muscles and the joint angles differed between crank quadrants, separate ANOVA’s for repeated measures (for the factor crank quadrant) were performed for each dependent variable. When a main effect of quadrant was identified, differences between quadrants were tested using a post-hoc Bonferroni test. Significant differences were assumed when *p* ≤ 0.05. All statistical analysis was conducted in SPSS for Windows (version 21.0, SPSS, IBM Inc, USA).

## Results

**Fig. 3:**
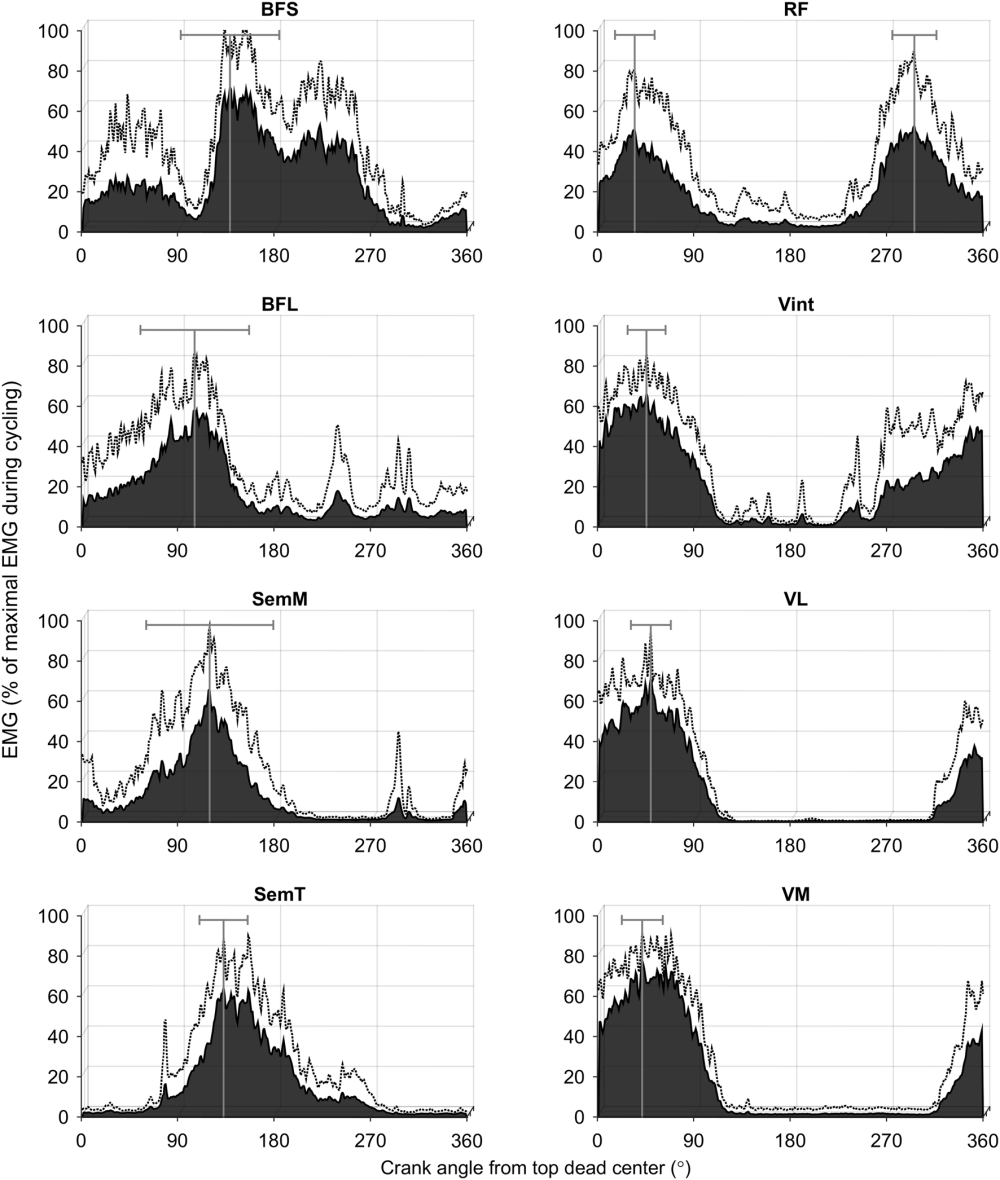
Normalized EMG (mean + SD) for biceps femoris short head (BFS), biceps femoris long head (BFL), semimembranosus (SemM), semitendinosus (SemT), rectus femoris (RF), vastus intermedius (Vint), vastus lateralis (VL), and vastus medialis (VM) muscles during the pedaling cycle. *Vertical lines* represent the *angles* at which peak EMG occurred (mean ± SD) for each muscle of all subjects

The normalized RMS data for each muscle are presented in Fig. 3.

**Fig. 4:**
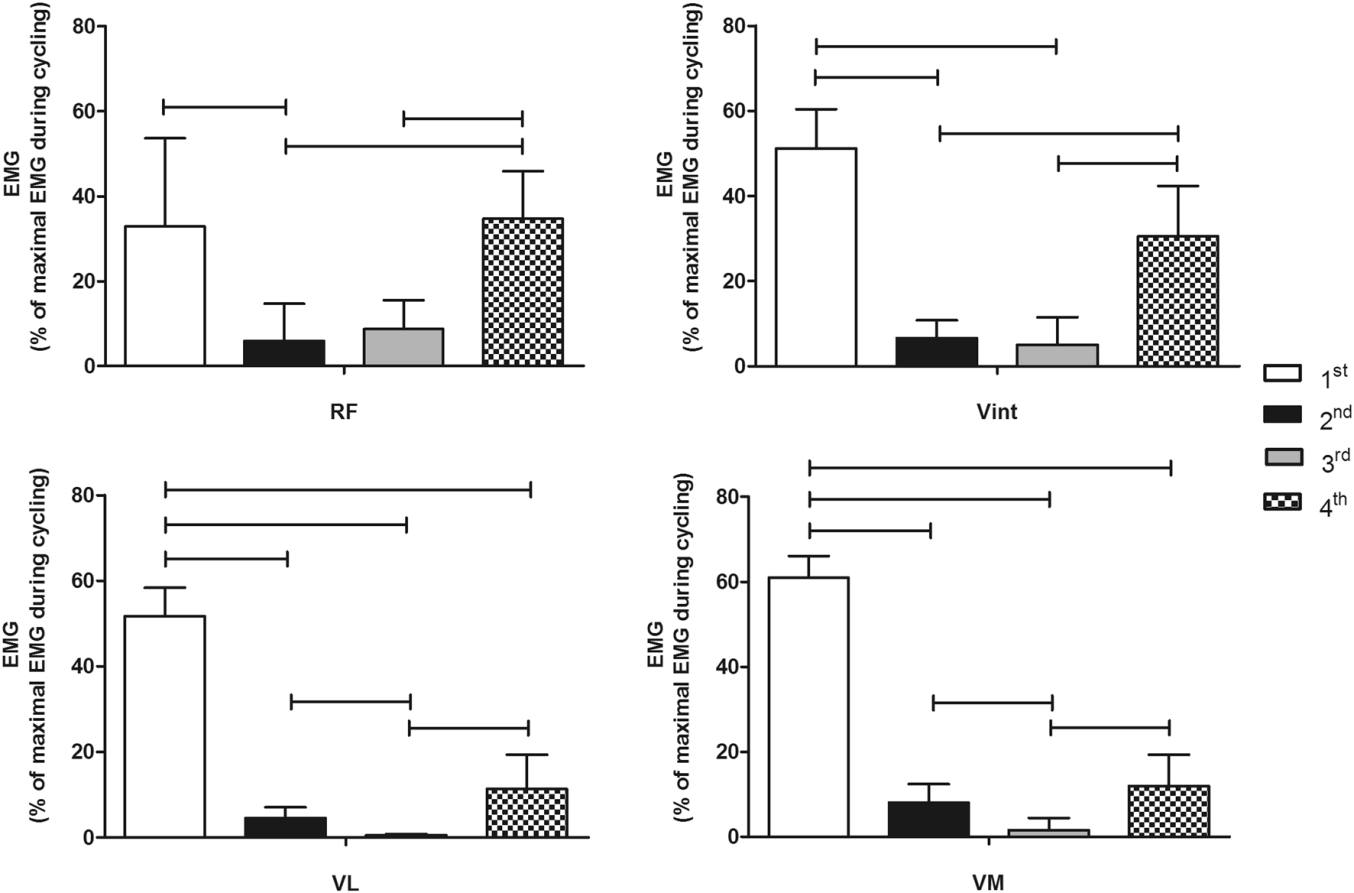
Normalized EMG activation (mean ± SD) for rectus femoris (RF), vastus intermedius (Vint), vastus lateralis (VL), and vastus medialis (VM) during the four quadrants (1st, 2nd, 3rd, and 4th) of the pedaling cycle. Statistically significant differences between quadrants are indicated by *lines* (*whiskers*), *p* ≤ 0.05

**Fig. 5:**
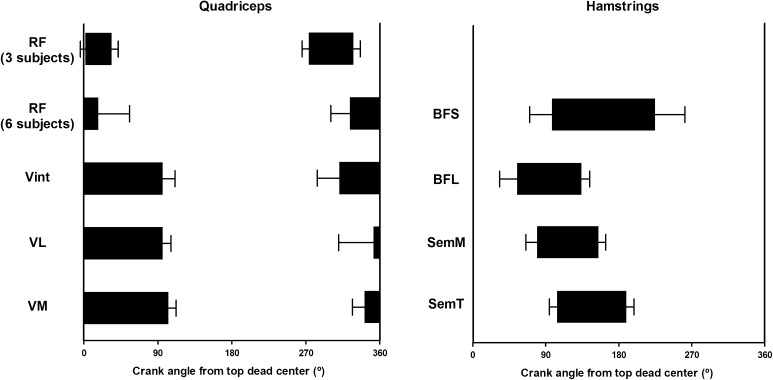
Onset and offset (mean ± SD) of quadriceps and hamstring muscle activity based upon crank angle from top dead center (TDC)

All four quadriceps muscles varied their activation level between quadrants (*F*_df, error_ was *F*_3, 24_ = 241.6 for VL, *F*_3, 24_ = 315.0 for VM, *F*_1.5, 12.2_ = 51.8 for Vint, and *F*_1.7, 13.8_ = 12.1 for RF). The vastii all showed their highest activation during the 1st quadrant. The VL and VM muscles showed a higher activation during the 1st (VL: 51.7 ± 6.7 %; VM: 61 ± 5.1 %) compared to all other crank quadrants (*p* < 0.01; Fig. 4). Vint was the only vastus muscle with no significant difference in activation between the 1st (51.2 ± 9.2 %) and 4th (30.5 ± 11.9 %) quadrants (*p* = 0.079; Fig. 4), indicating an earlier increase in activation at the beginning of the 4th crank quadrant compared to the VL and VM (Fig. 3). The VL and VM muscles also showed significantly greater activation in the 2nd compared to the 3rd quadrant (*p* < 0.01; Fig. 4). Vasti muscles had their EMG onset during the 4th crank quadrant (VL = 352 ± 43°; VM = 341 ± 15°, and Vint = 310 ± 27°) and offset at the beginning of the 2nd crank quadrant (VL = 96 ± 10°; VM = 103 ± 9° and Vint = 96 ± 15°; Fig. 5).

The bi-articular RF was the only quadriceps muscle to show two distinct activation bursts and these were in the 1st and 4th quadrants (Fig. 3) with no significant difference between them (32.9 ± 20.7 and 34.7 ± 11.1 %, respectively, *p* = 1.00; Fig. 4). The mean bursts in the 1st and 4th quadrants were significantly higher than activation during the remaining pedal cycle (*p* < 0.01; Fig. 4). The RF presented two patterns of activation. A continuous burst was found in six subjects between 4th and 1st crank quadrants (onset = 286 ± 47°; offset = 34 ± 76°). However, in three subjects, a double burst was seen in the 4th crank quadrant (onset = 273 ± 16°; offset = 329 ± 17°) and the 1st crank quadrant (onset = 1 ± 12°; offset = 64 ± 16°; Fig. 5).

**Fig. 6:**
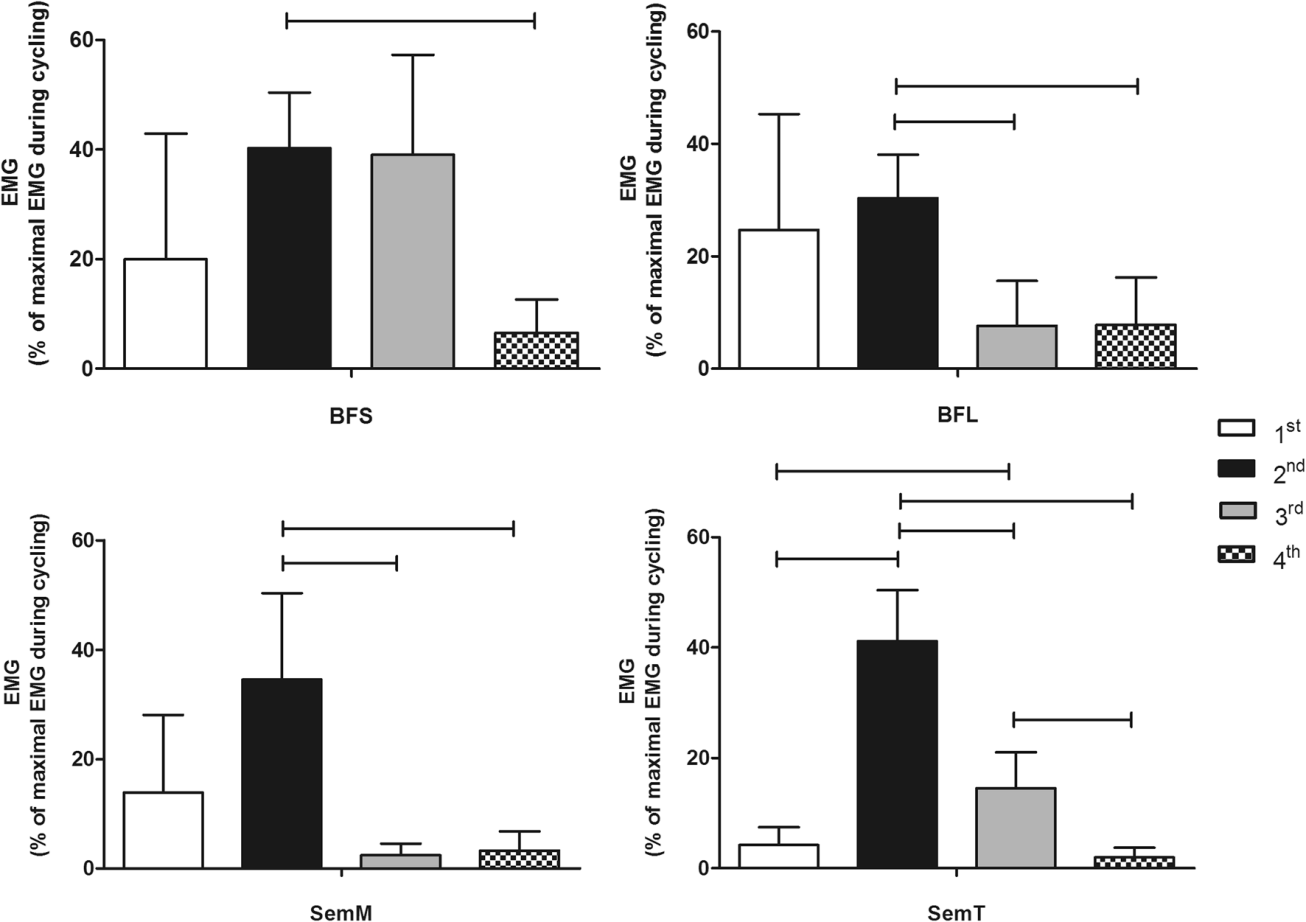
Normalized EMG activation (mean ± SD) for biceps femoris short head (BFS), biceps femoris long head (BFL), semimembranosus (SemM), and semitendinosus (SemT) during the four quadrants (1st, 2nd, 3rd, and 4th) of the pedaling cycle. Statistically significant differences between quadrants are indicated by *lines* (*whiskers*), *p* ≤ 0.05

All four hamstring muscles varied their activation level with quadrant (*F*_3, 12_ = 4.9 for BFS, *F*_1.7, 13.7_ = 9.2 for BFL, *F*_1.8, 14.2_ = 16.7 for SemM, and *F*_1.8, 14_ = 111.3 for SemT). All hamstring muscles showed their highest activation levels during the 2nd quadrant. A significant difference was observed in the BFS between the 2nd (40.2 ± 10.2 %) and 4th (6.5 ± 6.1 %) quadrants (*p* = 0.049; Fig. 6), with some activation also occurring in the 1st crank quadrant (Fig. 3). The BFL had a higher activation during the 2nd (30.5 ± 7.7 %) compared to both the 3rd (7.6 ± 8 %) and 4th (7.8 ± 8.5 %) quadrants (*p* < 0.01; Fig. 6) with activity increasing from TDC and decreasing directly after the 2nd quadrant (Fig. 3). The BFS onset occurred during the 2nd crank quadrant (297 ± 55°) with its offset in the 3rd crank quadrant (225 ± 74°). However, the BFL onset (54 ± 43°) and offset (134 ± 20°) were found between the 1st and 2nd crank quadrants (Fig. 5).

The SemM showed significant differences between the 2nd (34.5 ± 15.8 %) quadrant and both the 3rd (2.1 ± 2.1 %) and 4th (3.2 ± 3.5 %) quadrants (*p* < 0.01; Fig. 6). Similarly, the SemT muscle showed a significant difference between the 2nd (41.1 ± 9.3 %) and all other quadrants (*p* ≤ 0.05 for all comparisons; Fig. 6). Activity in the SemM began slightly earlier in the pedal cycle than in the SemT (Fig. 3). The SemM onset was during the 1st crank quadrant (79 ± 28°) with its offset in the 2nd crank quadrant (155 ± 18°) and the SemT onset was observed during the 2nd crank quadrant (104 ± 19°) with its offset at the beginning of the 3rd crank quadrant (190 ± 19°), see Fig. 5.

**Fig. 7:**
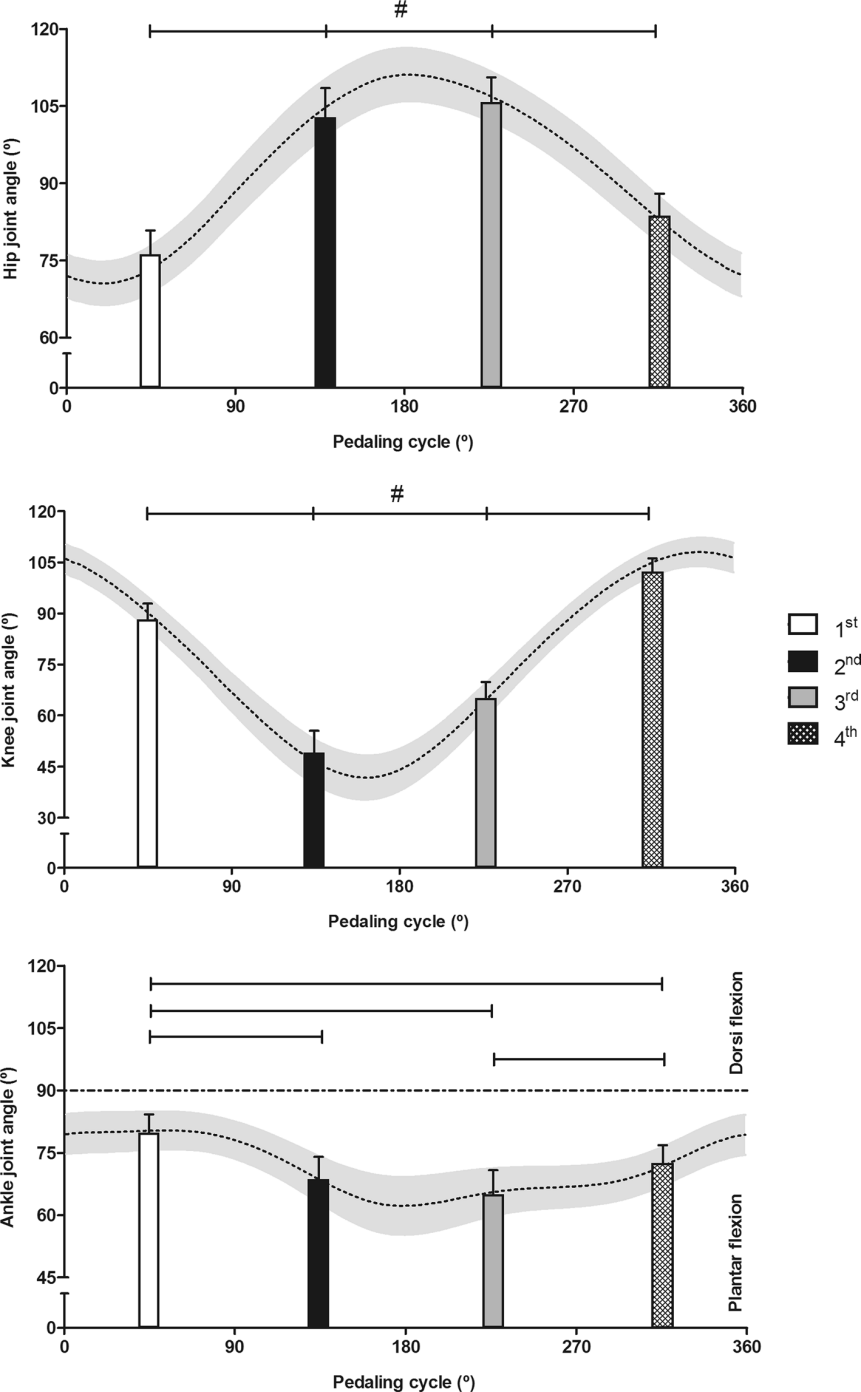
Mean (±SD) angles for hip, knee, and ankle joints during the pedaling cycle (*dotted lines*) and for respective quadrants (1st, 2nd, 3rd, and 4th) of the pedaling cycle (*bars*). # statistically significant differences between all quadrants for hip and knee joints, *p* ≤ 0.05. For the ankle joint, the statistically significant differences between quadrants are indicated by *lines* (*whiskers*), *p* ≤ 0.05

To relate activation patterns to leg joint configurations, kinematic data are presented in Fig. 7.

## Discussion

This study described muscular coordination of eight thigh muscles during cycling using intramuscular EMG. This provided the unique opportunity for describing activation patterns of Vint and BFS, muscles which are difficult to access with surface EMG.

Previous studies have shown that the greatest activity for the VL and VM occurs during the propulsion phase, beginning midway through the 4th quadrant, while the knee is extending (Dorel et al. 2008; Ericson et al. 1985; Jorge and Hull 1986; Ryan and Gregor 1992; Van Ingen Schenau et al. 1992). Similarly, in this study, the VL and VM were activated from the middle of the 4th quadrant to a crank angle of about 100° with peak activity during the 1st quadrant (right column, Fig. 3). The Vint demonstrated a longer period of activation than seen for the VL and VM (Fig. 5). According to Dorel et al. (2008), the RF is active between 270° until the end of the 1st quadrant, which was similar to Jorge and Hull (1986), who, however, described RF activity as not terminating until about 120–130°. Previous studies analyzing RF activity using onset and offset analyses during low intensity cycling have reported an onset range between 230–296° and an offset range between 85–99° of crank angle (Baum and Li 2003; Dorel et al. 2008; Jobson et al. 2013; Rouffet et al. 2009). As opposed to the previous studies, this study described a different RF behavior with two types of activation bursts, both a continuous burst and a double burst pattern. Six subjects activated RF with a continuous burst between the beginning of the 4th crank quadrant and the 1st crank quadrant. Two shorter, distinct bursts were observed in three subjects, with one burst during the 4th crank quadrant and the second burst during the 1st crank quadrant (Fig. 5). The RF was the only quadriceps muscle to display this double burst of activity.

Ericson et al. (1985) showed variation in the activation patterns between different hamstring muscles, where the SemM and SemT were activated between 150° and 270°, while the BF (represented as a single muscle) was activated during the complete pedaling cycle except for a short period when the pedal passed TDC. In this study, the separate analysis of the BFS and BFL muscle heads showed that the BFS was mostly activated during the 2nd and 3rd crank quadrants, whereas the BFL was already activated in the 1st quadrant until the middle of 2nd crank quadrant (Fig. 5). The SemM was active from the end of the 1st crank quadrant until the 2nd crank quadrant and the SemT was activated between the beginning of the 2nd crank quadrant until the beginning of the 3rd crank quadrant (Fig. 5), which was consistent with the results presented by Jorge and Hull (1986) and Dorel et al. (2008). As opposed to the previous literature (Blake et al. 2011; Ericson et al. 1985), this study found that both the SemM and SemT had their activation peaks prior to BDC and only SemT was still (although decreasingly) active a short period after BDC. BFS and BFL activation peaked during the 2nd crank quadrant (left column, Fig. 3).

In this study, there was very little variation in ankle angle during the 1st quadrant (80 ± 4°; Fig. 7) followed by a significant plantarflexion in the 2nd quadrant (69 ± 6°). The triceps surae presumably activated during the downstroke, providing a rigid link for optimized force transfer from the thigh muscles to the pedal. According to the studies by Chapman et al., different lower leg muscles are activated at different crank angles and their recruitment strongly depends upon training level and also differs between cyclists and triathletes (Chapman et al. 2007, 2008). Some plantar flexors (peroneus longus, soleus, and tibialis posterior) have been shown to be recruited only during the downstroke phase, whereas gastrocnemius lateralis is activated during both the down- and up-stroke phases and tibialis anterior showed activation during 2nd, 3rd, and 4th quadrants (Chapman et al. 2006, 2008). These data correspond to tibialis anterior dorsiflexing the ankle and the gastrocnemius lateralis flexing the knee during the up-stroke.

Efficient muscle coordination is required to provide an effective force profile. In terms of the pedal cycle, the force generation achieved to propel the bicycle forward has been attributed predominantly to the mono-articular muscles (BFS, VL, Vint, and VM) during the propulsion phase, whereas bi-articular muscles (RF, BFL, SemM, and SemT) may assist in directing the pedal forces and redistributing net moments over the joints during this phase (Gregor and Conconi 2000). The RF demonstrated behavior attributed to bi-articular function, in that its activation during the up-stroke phase could be explained by its hip flexor function. Using surface multichannel recordings, Watanabe et al. (2015) demonstrated that the proximal and distal regions of RF were activated during different parts of the pedaling cycle, with proximal activation primarily between 180° and 225° crank angle and middle-distal activation between 270° and 90° of crank angle. This suggests that different regions in RF might contribute to combined knee extension and hip flexion torque (middle-distal region) and to isolated hip flexion torque (proximal region). The RF fine-wire electrodes in this study were located in the middle region of the muscle. The two patterns of RF activity were due to the activation in three subjects decreasing below 20 % of maximum during the 4th quadrant. One of these bursts was earlier and the other later than the single burst seen in the other six subjects. This modified pattern may result in a more effective muscle force production and these subjects may have been more experienced cyclists. This, however, still remains to be investigated.

Activation of the deep thigh muscles Vint and BFS has not previously been investigated during cycling. Deep muscles may be predominantly involved in joint stability rather than movement and it might, therefore, be assumed that the Vint and the BFS are active over a larger range of motion than the more superficial muscles. The deep Vint muscle appears to coordinate with the other quadriceps muscles (VM, VL, and RF) during cycling (Saito et al. 2015). No difference was found between the 1st and 4th quadrants for Vint. Vint coordinated with all quadriceps muscles in this study, but appeared to be activated over a larger range of movement than the more superficial muscles, suggesting that it may also have a stabilizing function about the knee.

During cycling, the BFS showed highest activation during the 2nd and 3rd quadrants, where the knee joint was most extended and during the extension-flexion transition. In this case, the BFS was presumably activated at a longer muscle length and in synergy with the BFL during the transition from extension to flexion. Both the BFL and BFS muscles share the same muscle insertion and probably also the same deep aponeurosis, providing an intimate synergistic relationship. In this study, the medial hamstring muscles (SemM and SemT) were most activated during the 2nd quadrant, which indicated that SemM and SemT were recruited together with BFL and BFS, and all these muscles became a synergistic group during pedaling. The BFS was active longer than the other muscles in this synergi. Knee flexion presumably places the vastii in a more beneficial force–length relationship for force production during the activation seen in the 1st quadrant, thus contributing to the maximum pedal force occurring at about 90° (Candotti et al. 2007; Ericson and Nisell 1988; Neptune et al. 2000).

To promote force transfer to the pedal, neuromuscular activation during pedaling has to take into consideration the fact that joint angles, and therefore, muscle action types, lengths, and lever arms change over the crank cycle. In this study, intramuscular EMG was used to verify muscle activation during cycling. This is the first study using this technique in hip and knee extensors and flexors during cycling. The activation patterns of deep muscles (Vint and BFS) could, therefore, be described. A limitation of this study was the small sample size, in which BFS activity was successfully measured (four subjects). Furthermore, no differentiation was made between cyclists with different performance levels, which may have resulted in more different activation patterns than only the RF described here. Future research should include data concerning intramuscular EMG of the dorsi- and plantar flexors and the pedal forces during the bicycle performance to incorporate these data in a comprehensive analysis of muscle contributions to cycling performance. Furthermore, thigh intramuscular activation patterns may vary at different workloads and in different body configurations, and these should also be investigated in future research.

## Conclusion

Muscular coordination in quadriceps and hamstring muscles was investigated and related to the crank angle during cycling at a constant workload. Vastii muscles had the highest activation during the 1st quadrant of the pedal cycle, whereas for all hamstring muscles’, peak activation occurred during the 2nd quadrant. In three subjects, the RF showed a unique behavior with two burst activation (in the 1st and 4th quadrants), with peak activity in the 4th quadrant. The study presented a unique analysis of deep mono-articular muscles (Vint and BFS) and showed that these activated together with the superficial muscles, but for a longer duration.

## Abbreviations

ANOVA: Analysis of variance
BDC: Bottom dead center
BFL: Biceps femoris long head
BFS: Biceps femoris short head
EMG: Electromyography
QTM: Qualisys track manager
RF: Rectus femoris
RMS: Root-mean-square
SemM: Semimembranosus
SemT: Semitendinosus
TDC: Top dead center
Vint: Vastus intermedius
VL: Vastus lateralis
VM: Vastus medialis
